# Supplementary material for: Synthetic Genitourinary Image Synthesis via Generative Adversarial Networks: Enhancing Artificial Intelligence Diagnostic Precision
Source: J Pers Med. 2024 Jun 30;14(7):703. doi: 10.3390/jpm14070703 (PMC11278131; doi:10.3390/jpm14070703)
Supplement: Supplementary file 1 [file jpm-14-00703-s001.zip › jpm-3047776-supplementary.pdf]

Journal of *Personalized Medicine*

Article

Synthetic Genitourinary Image Synthesis via Generative Adversarial Networks: Enhancing AI Diagnostic Precision

Derek J Van Booven [1], Cheng-Bang Chen [2], Sheetal Malpani [3], Yasamin Mirzabeigi [3], Maral Mohammadi [4], Yujie Wang [2], Oleksander Kryvenko [3], Sanoj Punnen [5] and Himanshu Arora [1,4,5,*]

[1] John P Hussman Institute for Human Genomics, Miller School of Medicine, University of Miami, Miami, Florida, USA
[2] Department of Industrial and Systems Engineering, University of Miami, Miami, Florida, USA
[3] Department of Pathology, Miller School of Medicine, University of Miami, Miami, Florida, USA
[4] Department of Pathology, University of Debrecen in Hungary, Hungary.
[5] Desai & Sethi Institute of Urology, Miller School of Medicine, University of Miami, Miami, Florida, USA
[6] The Interdisciplinary Stem Cell Institute, Miller School of Medicine, University of Miami, Miami, Florida, USA
* Correspondence: Himanshu Arora (Hxa287@miami.edu)

Author Contributions: HA was the chief investigator. DVB, and HA, designed the study and developed the protocol. SM, YM, and MM carried out the pathological analysis plan. HA and DVB coordinated the central modeling investigations. CBC developed Spatial quantification methods. CBC and DVB validated the quantifications. HA, DVB coordinated the data collection. DVB, CBC and HA interpreted the data. HA and DVB developed the first drafts of the manuscript. CBC, SM, MM, YW have accessed and verified all the data in the study. All authors had access to all the data reported in the study. All authors contributed to the review and amendments of the manuscript for important intellectual content and approved this final version for submission. The corresponding author had full access to all the data in the study and had final responsibility for the decision to submit for publication.

Competing Interest Statement: Disclosure of Patent Information: The authors wish to inform that the technology presented in this study is part of a provisional patent application that has been filed with the United States Patent and Trademark Office (USPTO). The application has been assigned Serial No. 63/598,207 and was filed on November 13, 2023. The patent application is currently pending. Some of the authors of this paper are listed as inventors in the patent application. This patent filing may constitute a potential conflict of interest, and this statement serves to disclose this relationship in the interest of full transparency.

Supplementary data

Supplementary Figure 1. Showing synthetic images of Bladdar.

Supplementary Figure 2. Showing synthetic images of Cervix.

Supplementary Figure 3. Showing synthetic images of Kidney.

Supplementary Figure 4. Showing synthetic images of Ovary.

Supplementary Figure 5. Showing synthetic images of Prostate.

Supplementary Figure 6. Showing synthetic images of Testis.

Supplementary Figure 7. Showing synthetic images of Vagina.

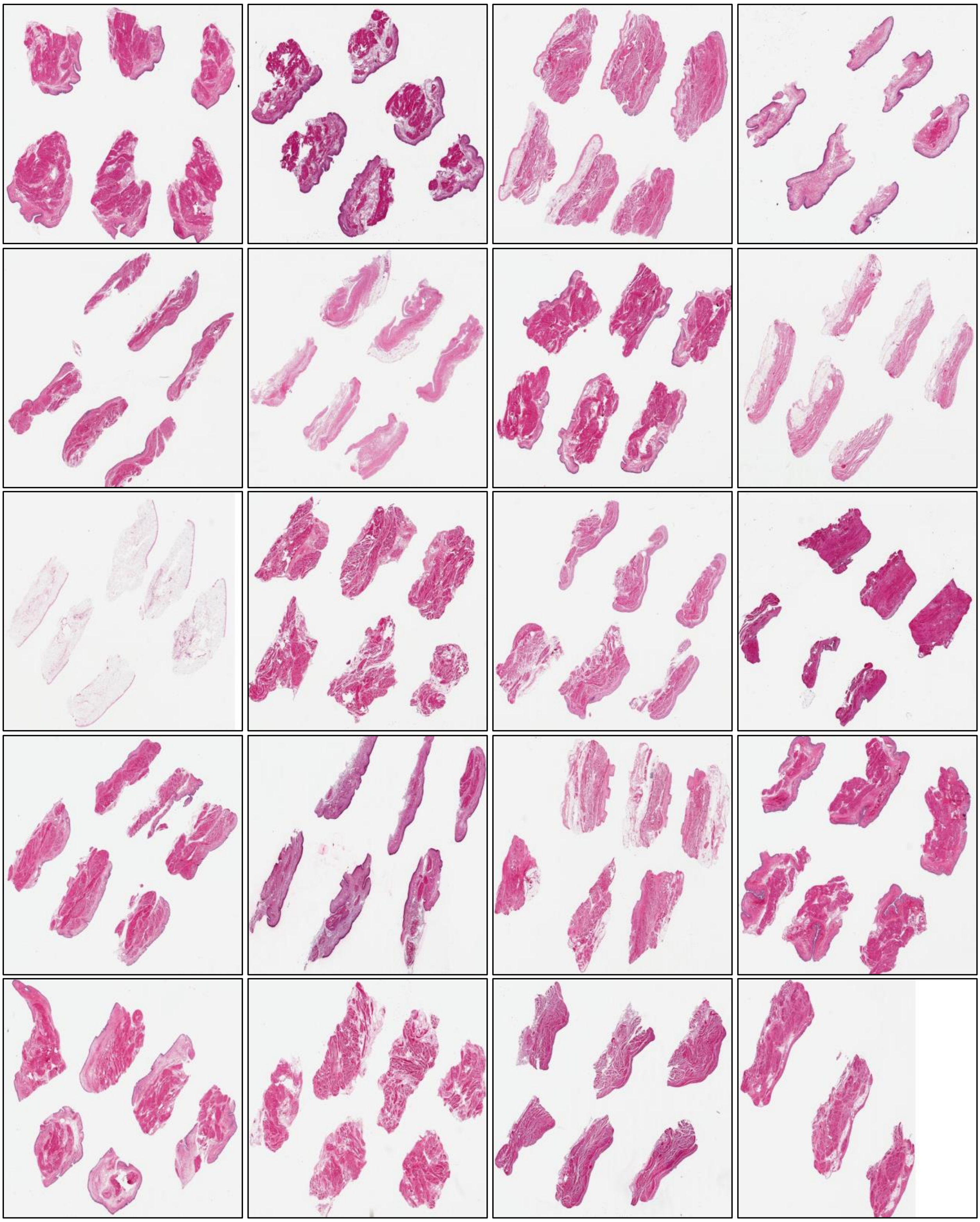

Supplementary Figure S1. Showing synthetic images of Bladdar.

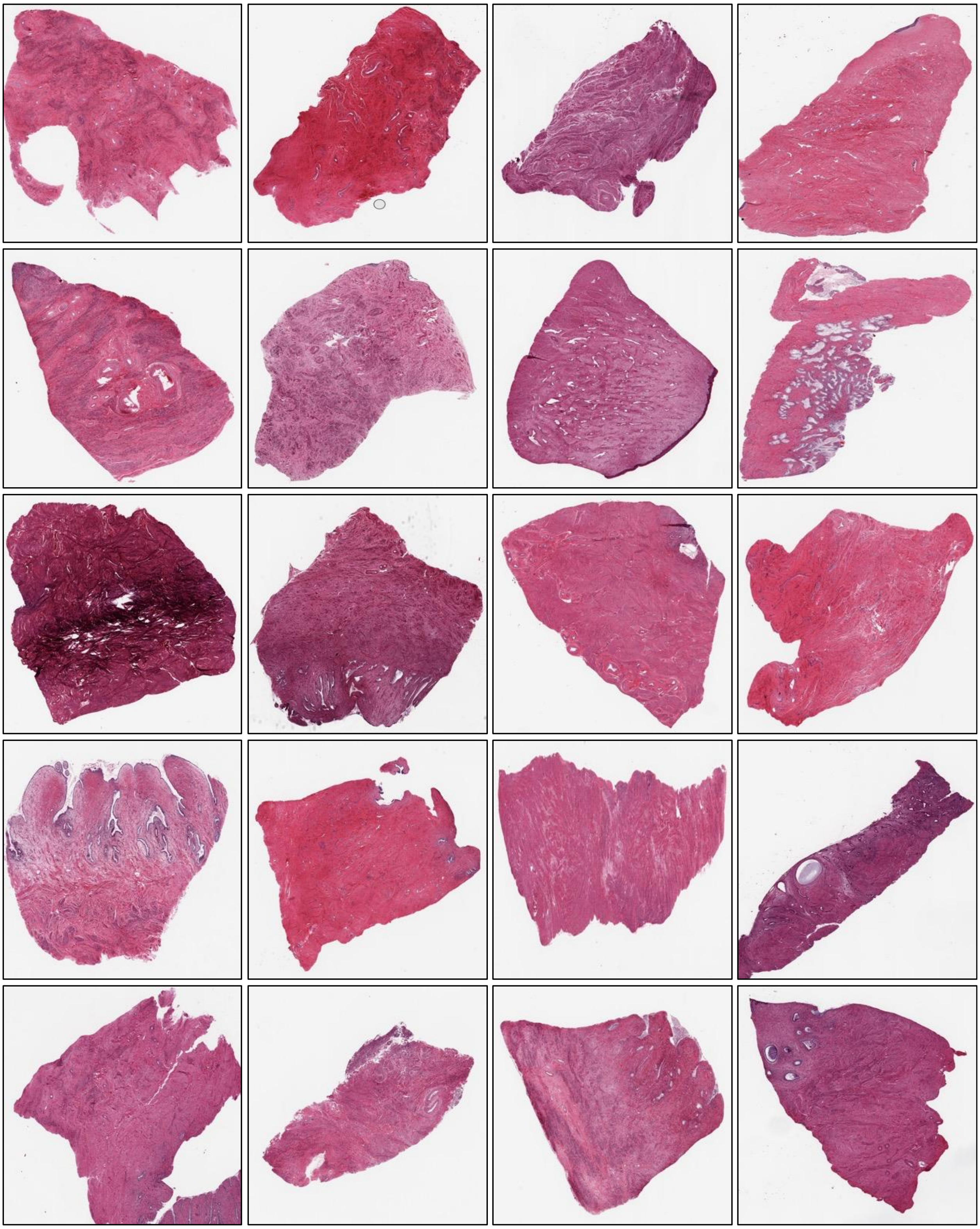

Supplementary Figure S2. Showing synthetic images of the Cervix.

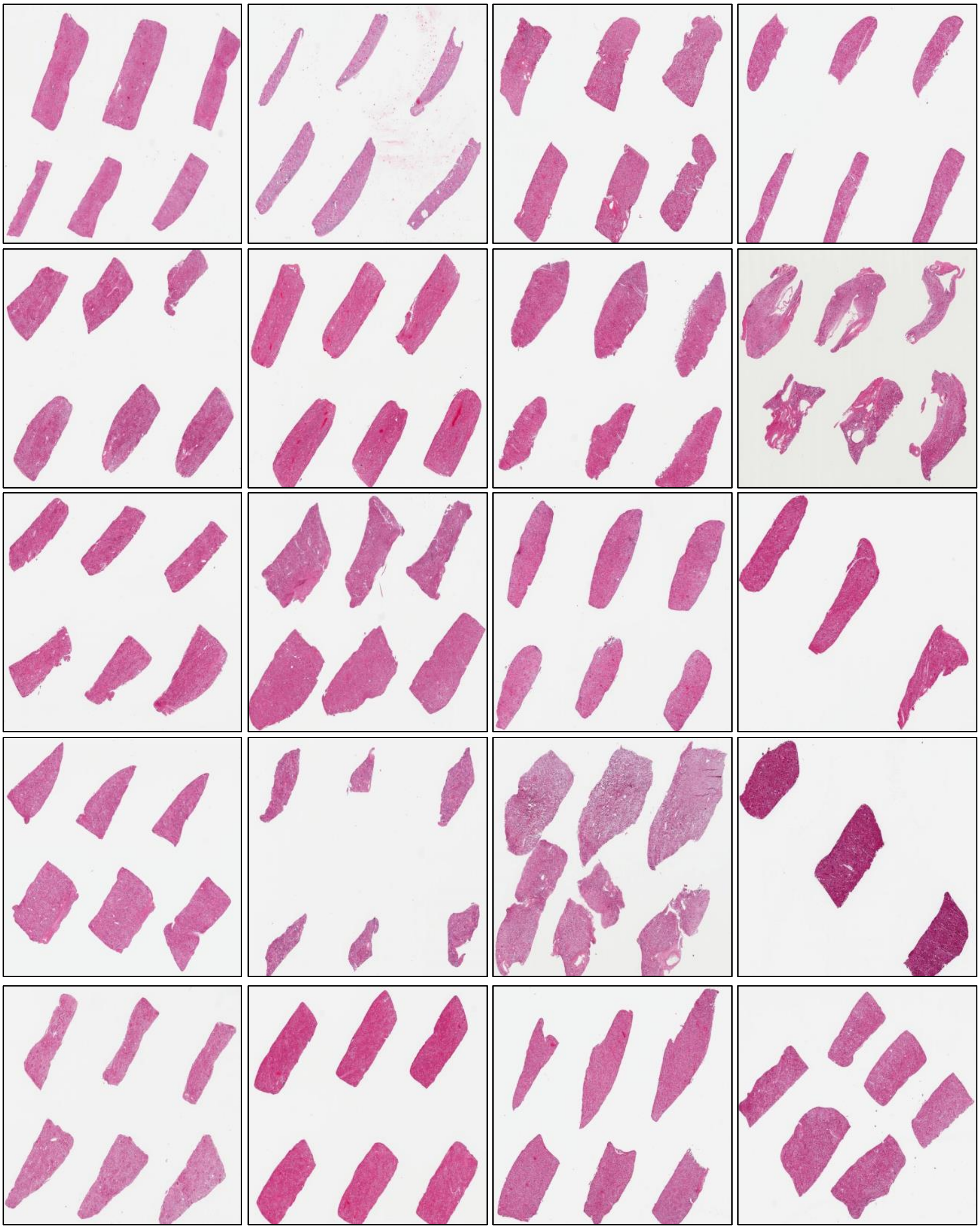

Supplementary Figure S3. Showing synthetic images of the Kidney.

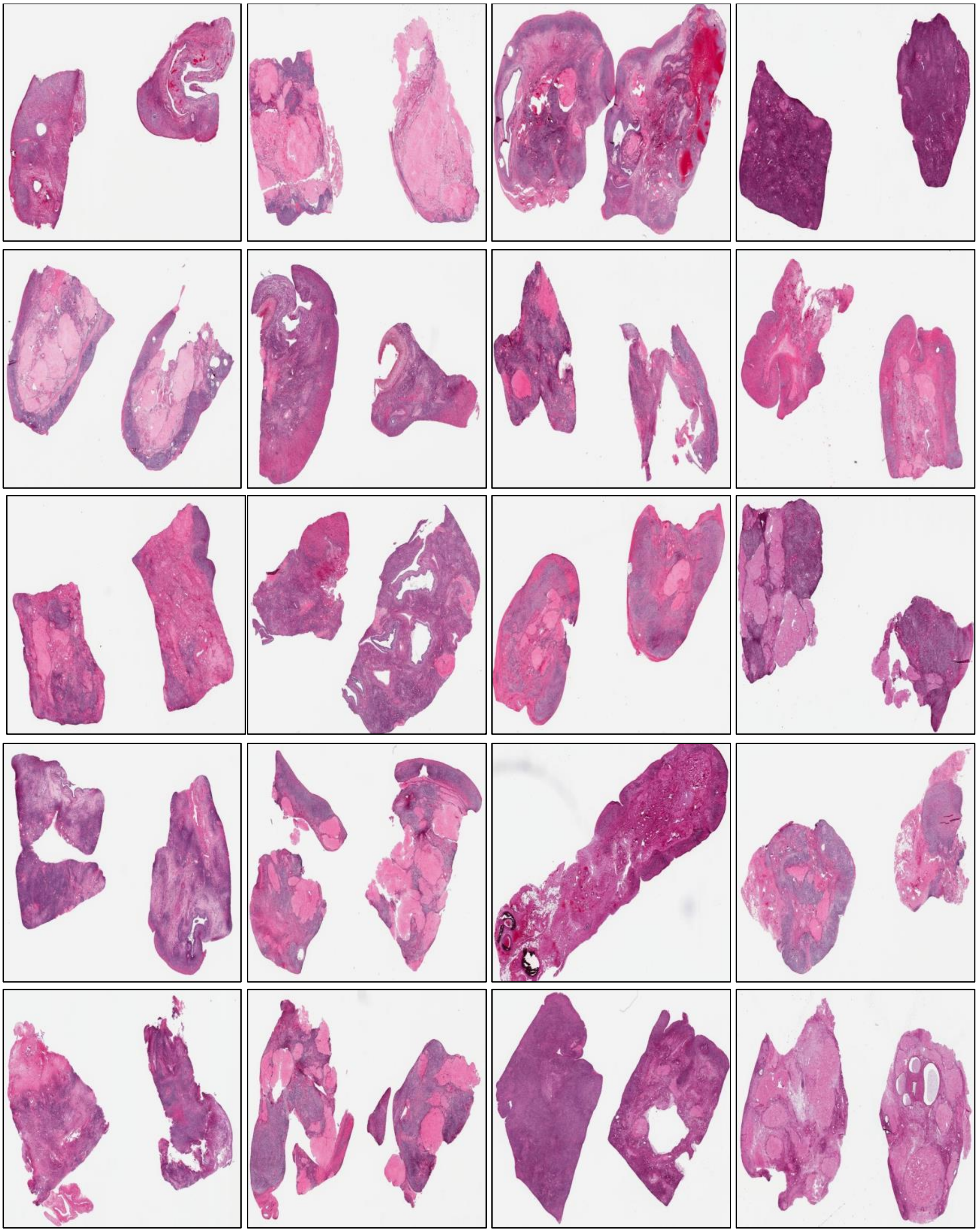

Supplementary Figure S4. Showing synthetic images of the Ovary.

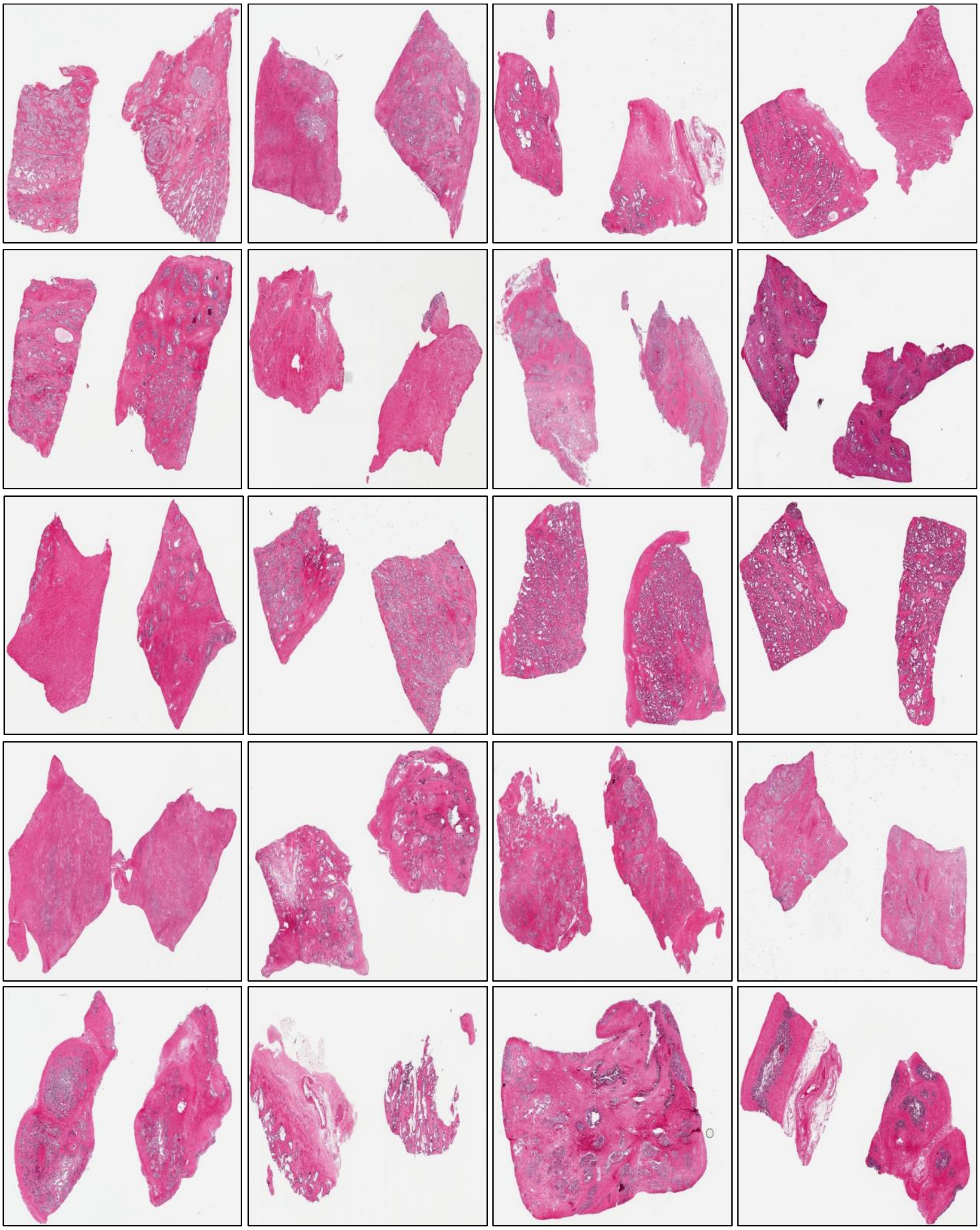

Supplementary Figure S5. Showing synthetic images of the Prostate.

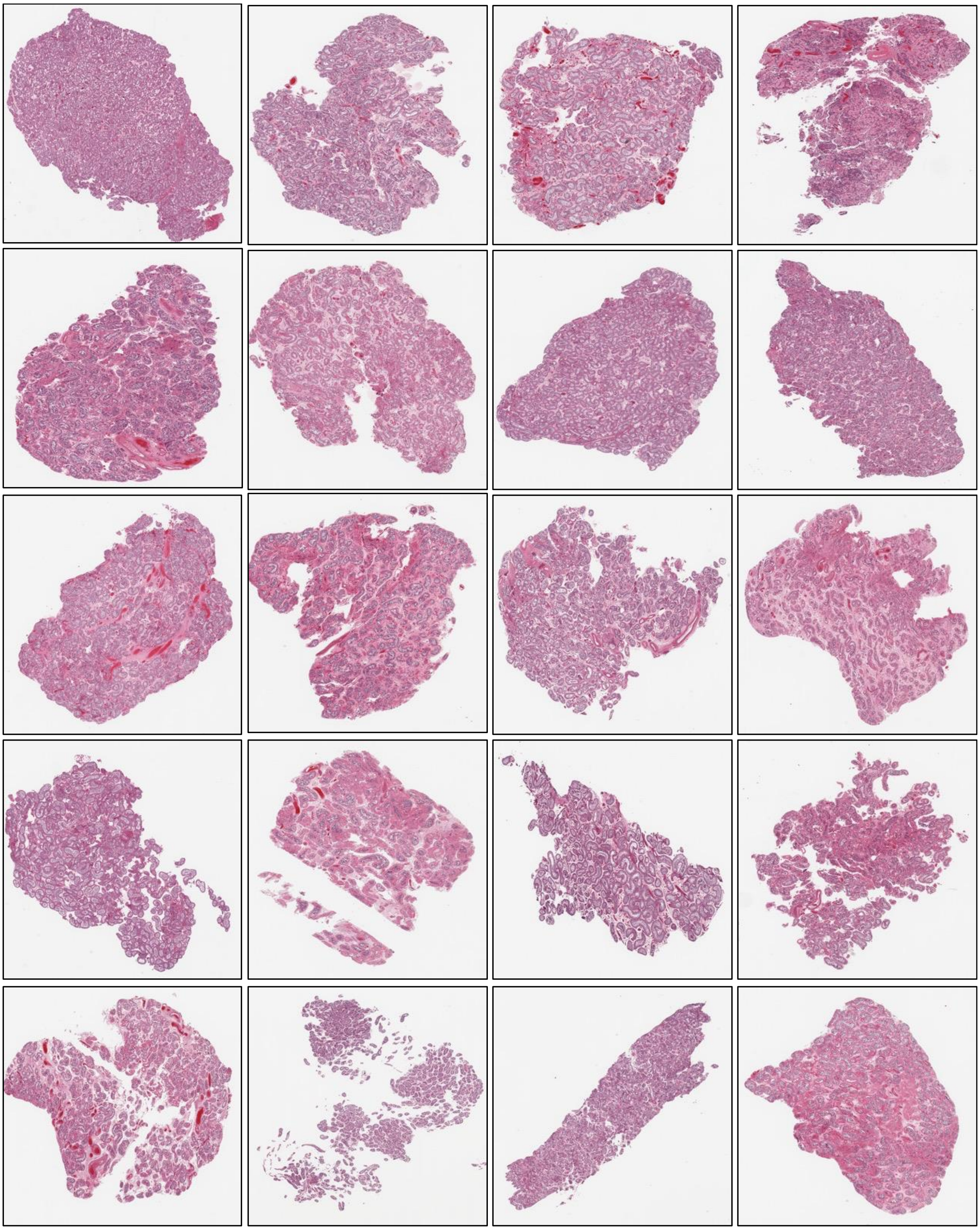

Supplementary Figure S6. Showing synthetic images of the Testis.

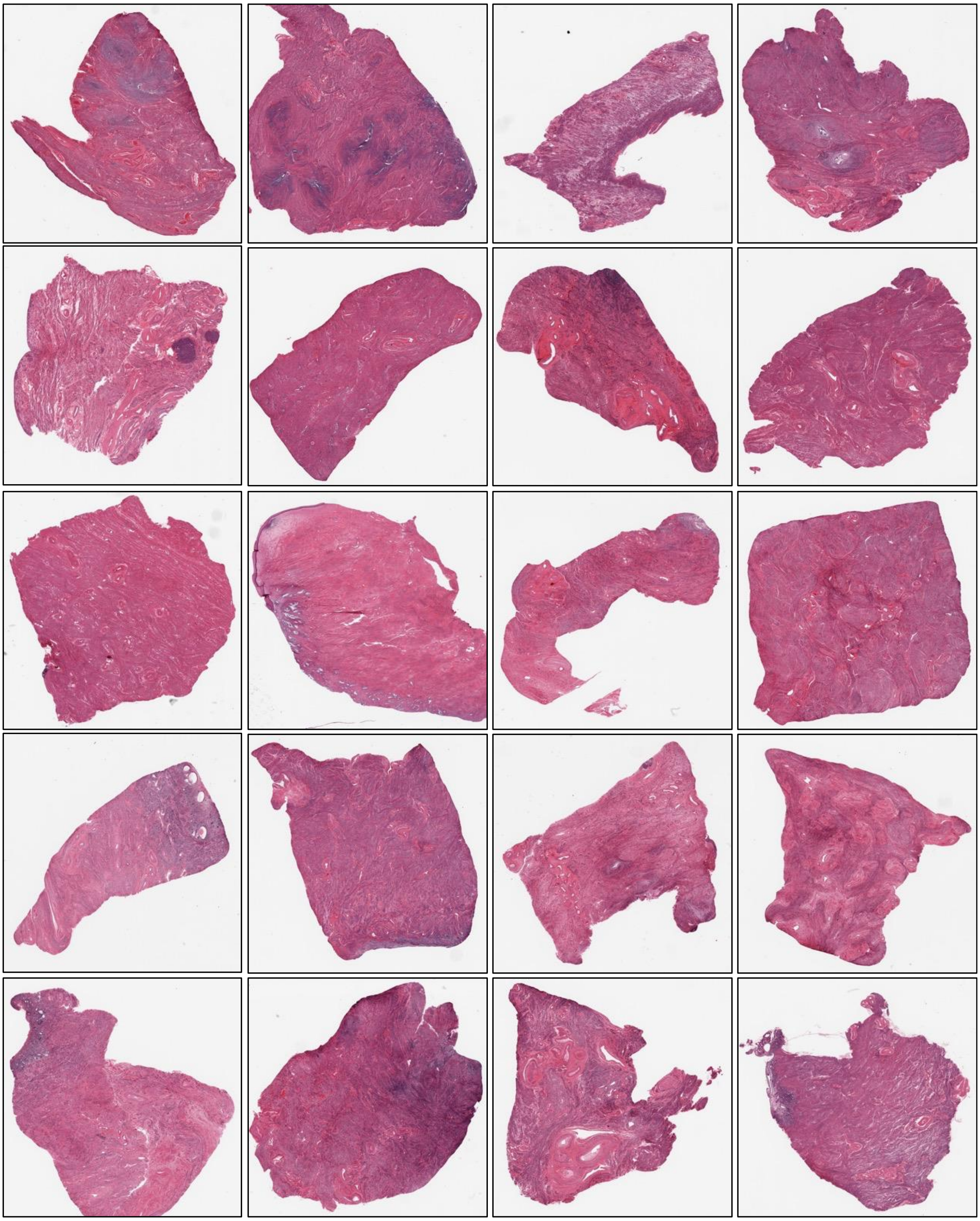

Supplementary Figure S7. Showing synthetic images of the Uterus.

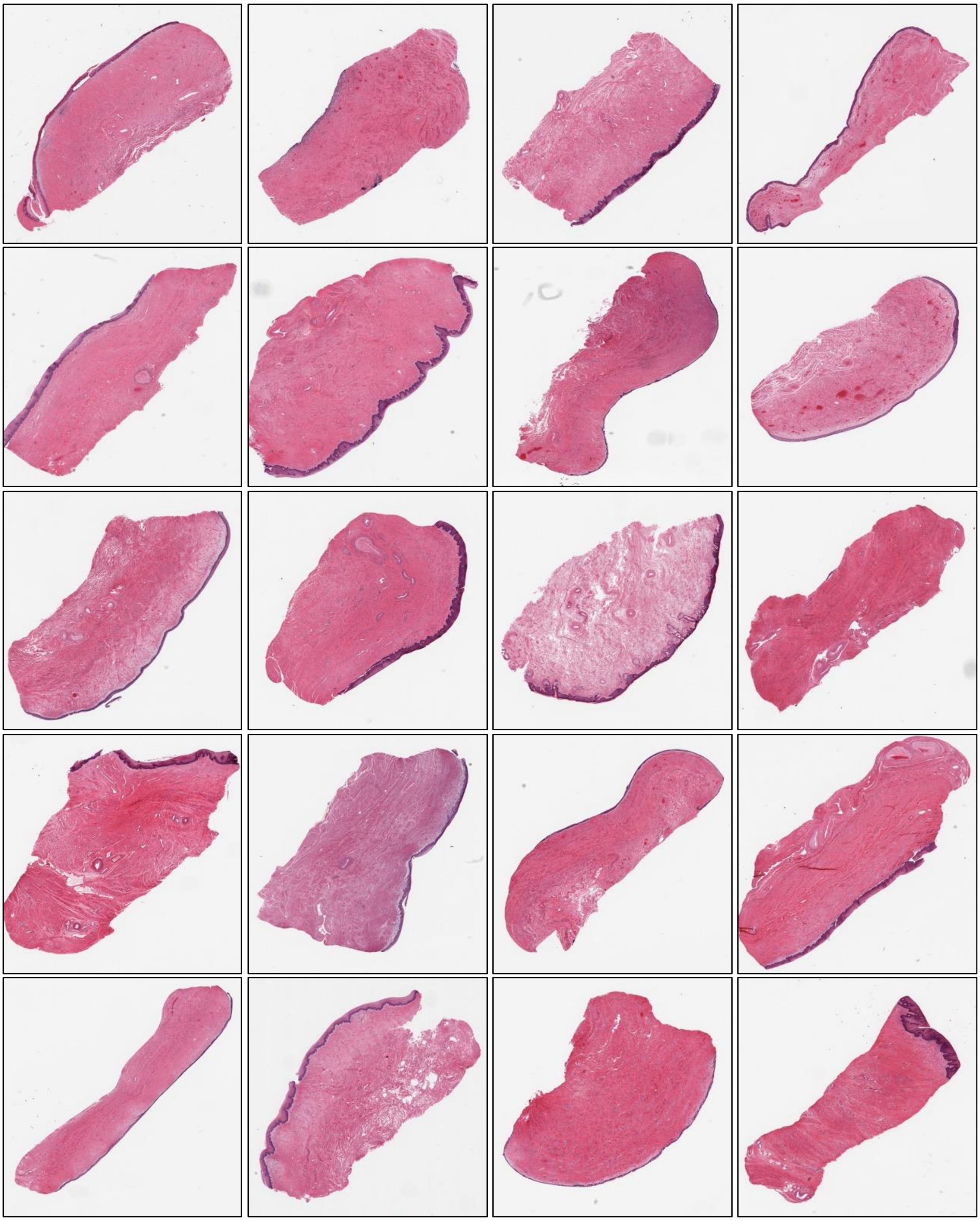

Supplementary Figure S8. Showing synthetic images of the Vagina.
